# Parenteral Medication Prescriptions, Dispensing and Administration Habits in Mongolia

**DOI:** 10.1371/journal.pone.0115384

**Published:** 2014-12-22

**Authors:** Gereltuya Dorj, Bruce Sunderland, Delia Hendrie, Richard Parsons

**Affiliations:** 1 School of Pharmacy, Bentley, Curtin University, Perth, Western Australia, Australia; 2 School of Public Health Curtin University, Perth, Western Australia, Australia; Universidad de Valladolid, Spain

## Abstract

High levels of injection prescribing were reported in Mongolia. Understanding the factors influencing the injection prescribing is essential to reduce their inappropriate use. The study evaluated the views, experiences and attitudes of community members associated with the prescribing of injections in Mongolia. A structured questionnaire focusing on respondents' characteristics, experiences and views about injections was developed and administered face-to-face to community members in Ulaanbaatar, Mongolia. Standard descriptive statistics were used to summarize demographic data and responses to the questionnaires. Dependant variables were compared using Kruskal-Wallis Tests for independence. Statistical analyses were performed using SPSS Version 21.0. Six hundred participants were approached and the response rate was 79% (n = 474). Almost half of the respondents were aged between 31 and 50 (n = 228, 48.1%) and 40.9% of respondents were male (n = 194). Most respondents were from Ulaanbaatar city (n = 407, 85.7%). All respondents had received injections in the past and 268 (56.5%) had received injection in the past year. The most common reason for having an injection in the past year was reported as treatment of a disease (n = 163, 60.8%), or for administration of vitamins (n = 70, 26.1%). Injections were prescribed by a doctor (n = 353, 74.9%), dispensed by a pharmacist (n = 283, 59.7%) and administered by a nurse (n = 277, 54.9%). Only 16% of all respondents had the expectation of receiving injections when they visited a doctor (n = 77). An important perception regarding injections was that they hastened the recovery process (n = 269, 56.8%). When asked their opinion about therapeutic injections, 40% of all respondents agreed that injections were a better medicine (n = 190) than oral medications, with older respondents strongly agreeing (p<0.001). Based on this total sample, approximately 1891 injections per 1000 patients were administered. The excessive injection use seems to be promoted by inappropriate prescribing, dispensing and administration of medication by doctors and others.

## Introduction

Injection medicines are commonly used in healthcare settings for the prevention, diagnosis, and treatment of various illnesses. Unsafe injection practises including the re-use of equipment in the absence of sterilization can place community members and healthcare providers at risk of infectious and non-infectious adverse events [1]. Factors giving rise to unnecessary parenteral medication prescribing in developing countries include socio-cultural, economic and structural factors. Studies from developing countries suggest that injections are overused particularly because of health practitioners' prescribing practises and community members' preference for injections over oral medications [2]–[6]. The belief in an injection as a strong tool for restoring and maintaining health is mutually supported by health professionals and community members in some developing countries [7]. Previous findings have suggested that patient demand may cause prescribers to prescribe and administer injections for patient satisfaction [8] [9], whereas in contrast others have indicated that community members were more open to alternatives to injections [10]. A study in Uganda and Indonesia which questioned the causes for injection prescribing reported that local belief about illness, concepts of efficacy, economic incentives for private or informal providers and lack of patient-provider communication were the main reasons [11]. A systematic review of studies from 13 developing countries regarding injection use and safety reported that in eight of those countries, 25–96% of outpatients visits resulted in at least one injection being prescribed, and for five countries a majority of the administered injections were unnecessary. Commonly administered parenteral injections included vitamins, antibiotics, analgesics and quinine [12]. Previous studies have reported inappropriate use of injections with respect to standard treatment guidelines in Mongolia [13], [14]. A later study has observed a reduction reporting eight injections per person per year (p<0.001) [15] however the small sample size (200) limits generalisation. Worldwide studies on hepatitis C prevalence reported wide range of estimates including 0.9% in India [16], 3.2% in China [17] to 22% in Egypt [18]. Substantial association between prevalent hepatitis C infection and unsafe therapeutic injections has been reported in previous studies [18]–[20]. The World Health Organization (WHO) has estimated that unsafe injections accounted almost two million of hepatitis C infections in 2000 [21]. Given the high prevalence of antibody hepatitis C (anti-hepatitis C) in Mongolia (16%–24%) [22], it is important to minimise unnecessary injection practises in the country especially on public health grounds.

In Mongolia, the pharmaceutical procurement sector is 100% privatized. Drugs are distributed through organizations such as drug wholesalers and retail drug outlets (community pharmacies and revolving drug funds). Recent statistics show there were 703 community pharmacies, 75% of which had one to two branches in Mongolia [23]. The Health Insurance Fund a single national fund with 80% of the population insured, finances a wide range of hospital care and outpatient medical expenses including 107 drugs in the Essential Drugs List of Mongolia [24].

## Objective

To evaluate community views, knowledge, attitudes and experiences of community members associated with prescribing injections in Mongolia and to assess other factors that may promote injection overuse in Mongolia

## Methodology

### Development of the questionnaire

The development of a questionnaire was based on the World Health Organization (WHO) developed guide: Injection Practises: Rapid Assessment and Response Guide [25] and other research findings [1], [2], [10], [11], [26], [27].

A 33-item structured questionnaire asked general questions regarding frequencies of injections use, use of injections in the past, experiences and views about a consultation in the past year and previous ones, knowledge about safe injection use and attitudes about injections versus other administration routes. However, all specific injection use data were focused on community members' encounter with a health care provider in the past year.

### Validation of the questionnaires

Two actively working professional translators with more than 15 years of experience and whose native language was Mongolian completed the English to Mongolian, and back translations to assure accuracy and minimize any possible bias. These translators were unknown to each other [28]. The author made adjustments resulting from any inconsistencies. For content and construct validity of the questionnaire, a pilot study was completed. Forty community members in a selected hospital waiting area were requested to complete the questionnaire of which 25 agreed. These were analysed for validity and clarity. Modifications regarding some wording terms and sequencing of the questions were made after the pilot study, in order to improve the completeness and clarity of questions. No major omissions were identified. These responses were not used further in the study.

### Selection of respondents

As recommended in the guide [1], a sample of community members, who were confirmed to be at least 18 years of age, was selected by administering the questionnaire face-to-face at pre-determined public locations to obtain samples from different socio-economic groups. Questionnaires were administered at 55 different locations. These included three public central hospitals in urban and five district hospitals in semi-urban districts; five Family Group Practices (FGPs) located in urban and 15 semi-urban districts; three private hospitals in urban and semi-urban districts; one university in urban and two in semi-urban districts; three supermarkets in the city centre and 19 small shops in the semi-urban areas.

### Questionnaire administration

A community member information sheet, written in Mongolian, was issued to potential respondents and the nature of the questionnaire was explained by the researcher. Prior to administering the questionnaire, a verbal consent was obtained because the participation was on a volunteer basis and all participants were de-identified. Most of the questionnaires were completed by participants. In some cases, however, the researcher administered the questionnaire to the participant verbally and completed the questionnaire based on their responses. The survey took place in public quiet areas, for example hallways of hospitals, universities or waiting areas in supermarkets, whenever possible. All questionnaires were administered during the winter period associated with a high prevalence of acute respiratory infections (January-March), 2010 in Ulaanbaatar, Mongolia.

### Data analysis

Data from the questionnaires were entered into Microsoft Excel© for basic analysis. The statistical analysis was completed using the Statistical Package for Social Sciences (SPSS Version 21.0). Standard descriptive statistics were used to summarize demographic data and responses to the questionnaires (frequencies for categorical variables, means and standard deviations for variables measured on a continuous scale). Questions were coded as 1- Yes, 2- Sometimes, 3 – No. Dependant variables were compared by a Kruskal-Wallis Test for independence. The differences between individual groups were identified by performing a pairwise comparison. A p value of <0.05 was considered to be statistically significant.

### Ethical consideration

The Human Research Ethics Committee of Curtin University, Western Australia approved the study protocol, including the consent procedure (PH-11-2010).

## Results

Six hundred community members aged over 18 years were contacted at various locations (pharmacies, shopping centres, hospitals and universities) in Ulaanbaatar, Mongolia. Of these 474 agreed to complete the questionnaire, giving a response rate of usable questionnaires of 79%. Non-respondents included mostly people from the younger age group (18–30 years), who refused to participate when asked and those who agreed but were unable to complete the questionnaire. Almost half of the respondents were aged between 31 and 50 years (n = 228, 48.1%), 40.9% were male (n = 194), and their average income converted into US dollars was US$193 per month (n = 99, 20.9%). In addition, for comparison purposes, relevant census data are provided for Mongolia (Table 1).

**Table 1:** Demographic characteristics of respondents.

Variables	Study, N = 474, n (%)	Census data of	*p* Value
		Mongolia, 2011	
Age (years)			<0.0001
18–30	198 (41.8)	586,302 (35.6)	
31–50	228 (48.1)	746,834 (45.3)	
≥51	48 (10.1%)	315,188 (19.1)	
Gender:			0.0003
Male	194 (40.9)	937,271 (49.2)	
Female	280 (59.1)	968,698 (50.8)	
Marital status:			<0.0001
Single	148 (31.2)	344,679 (20.9)	
Married	250 (52.7)	1,140,111(69.2)	
Divorced	30 (6.3)	35,329 (2.1)	
Separated	25 (5.3)	23,576 (1.4)	
Widowed	21 (4.4)	104,629 (6.3)	
Education:			0.0004
Higher	116 (24.5)	392,572 (20.6)	
Secondary	238 (50.2)	869,240 (45.6)	
Primary	98 (20.7)	562,485 (29.5)	
Other	22 (4.6)	81,672 (4.3)	
Occupation:			0.0994
Employed	247 (52.1)	911,664 (66.2)	
Unemployed	58 (12.2)	164,116 (11.9)	
Civil servant	66 (13.9)	-	
Student a	74 (15.6)	300,494 (21.8)	
Military servant	29 (6.1)	-	
Monthly income (MNT) c:		379.400 b	-
<90,000	83 (17.5)		
91,000–200,000	77 (16.2)		
201,000–300,000	99 (20.9)		
301,000–400,000	90 (19.0)		
401,000–500,000	68 (14.3)		
>501,000	57 (12.0)		

a Economically non active population.

b Average income in 2011 in Mongolia.

c MNT- Mongolian National Tugrug (currency), equivalent to 1300 USD at the time of the study.

- No data were available.

A comparison of the sample of community members with population data [23] indicated statistically significant differences with respondents being younger and the sample comprising more females, more singles and separated people and having higher education levels than the Mongolian population. Most respondents were from the Ulaanbaatar region (n = 407, 85.7%) where the survey was administered.

### Injection exposure

Data collected on the nature and prevalence of injection use revealed that all respondents had received at least one injection in the past and 268 (56.5%) had received injections in the past twelve months.

**Figure 1:**
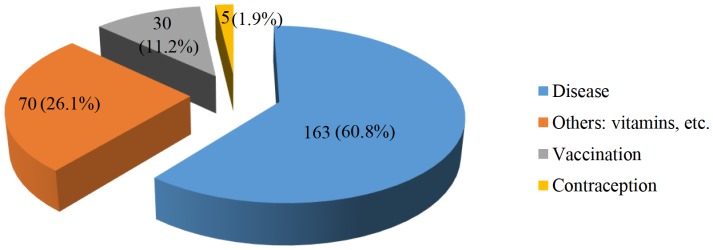
Community members' stated reasons for being given an injection.

All respondents reported that the most common reason for having an injection in the past twelve months was for treatment of a disease (n = 163, 60.8%), for administration of vitamins (n = 70, 26.1%), and some had injections for vaccinations and contraception (Fig. 1).

Injections were commonly reported for the management of symptoms of weakness, respiratory symptoms, which included cough, sore throat or pneumonia.

To further explore the extent of received injections, respondents were asked to indicate the number of injections they had for their last treatment. Of the 163 participants, who had injections for treatment of a disease, over 80% (n = 137) had between one and four injections and almost 16% (n = 26) reported five or more injections. A single injection was usually given for vaccination and always for contraception (Table 2).

**Table 2:** Reason and number of injections received by respondents for treatment occuring in the past year.

Reason of injection/Number of injections a	One	2–4	5–8	>8	Injection exposure per 1000 of all respondents per year
	n(%)	n(%)	n(%)	n(%)	
Disease	59(36.2)	78(47.8)	15(9.2)	11(6.7)	1055
Vaccination	29(96.7)	1(3.3)	-	-	67
Contraception	5(100)	-	-	-	11
Others: vitamins, etc.	40 (32.5)	67(54.5)	12(9.7)	4(3.3)	758

a Respondents could select more than one option.

### Quality of care

In terms of using new needles and syringes, a majority of all respondents was aware of these requirements and only 39 respondents (8.2%) said they did not know.

Questions regarding unwanted effects of injections in the past were presented and about 20% of all respondents (n = 91) had one of the proffered side effects after previous injections. Of this group, similar proportions experienced a warm feeling under the skin (n = 23, 20.9%) or a swollen or hard lump under the skin (n = 26, 23.6%). Less common was extravasation and experiencing fainting after having an injection.

**Figure 2:**
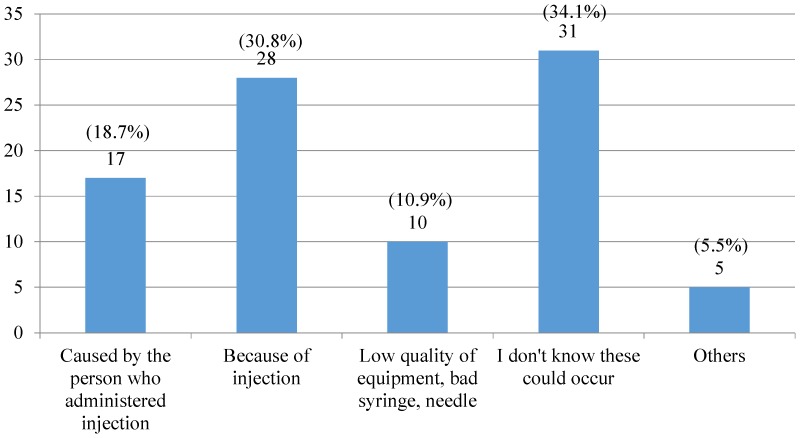
Possible reasons for side effects occurring from an injection for those who experienced a side effect.

When presented with reasons regarding side effects from injections, several possible options were put forward in the questionnaire. About one-third (n = 31, 34.1%) did not know that these effects could occur from an injection whereas others attributed them to the injection or the injection techniques employed (Fig. 2).

Of those experiencing side effects from injections approximately one-third consulted a doctor (n = 30, 32.9%) and others went to hospital (n = 15, 16.7%) or consulted a pharmacist (n = 6, 6.3%). However, almost one-half of respondents did not do anything (n = 40, 44.0%), which may be due to respondents not recognizing that those symptoms were side effects related to an injection or considering them minor.

### Characteristics of prescribers, suppliers and administrators of injections

#### Injection prescribers and suppliers

The current guidelines for ambulatory care specify that patients who need an injection should be referred to a hospital [29]. In Mongolia, most drugs can be purchased, including injections over-the-counter (OTC) [14], [30]. Therefore, all participants were asked about prescribers and suppliers of the last injections administered to gain an insight to this practice. The main prescribers were doctors (75% to 92%), who were legal prescribers. Other practitioners were less frequently sought for prescribing/selling injections and such provision is illegal under current regulations [29]. Of the 474 respondents, most (n = 353, 74.9%) obtained their injections on prescription with most being dispensed from pharmacies (n = 283, 59.7%). Pharmacists occasionally prescribed and supplied OTC injections according to 5 to 22% of respondents. It is noteworthy that nurses prescribed at a similar frequency. Doctors illegally supplied injections to between 25 and 40% of respondents which is an illegal practise, except in an emergency situation or as an inpatient (Table 3).

**Table 3:** Prescribers and suppliers of injections.

Category a	Injection prescribers			Injection suppliers/dispensers		
	Yes	Sometimes	No	Yes	Sometimes	No
	n (%)	n(%)	n(%)	n(%)	n(%)	n(%)
Doctor	353 (74.9)	75 (15.9)	43 (9.1)	118 (25.0)	69 (14.6)	285 (60.4)
Pharmacist	24 (5.1)	79 (16.7)	370 (78.2)	283 (59.7)	71 (15.0)	120 (25.3)
Nurse	30 (6.4)	66 (14.0)	376 (79.7)	21 (4.4)	54 (11.4)	397 (84.1)
Traditional practitioner/Seller	35 (7.4)	64 (13.6)	373 (79.0)	31 (6.5)	50 (10.6)	391 (82.8)

a Some responses were missing for each category.

It was evident that pharmacists dispensed/supplied the majority of injections with or without a prescription. Approximately 15% of respondents stated that injections were supplied each by nurses and traditional practitioners (Table 3).

#### Administration of therapeutic injections

**Figure 3:**
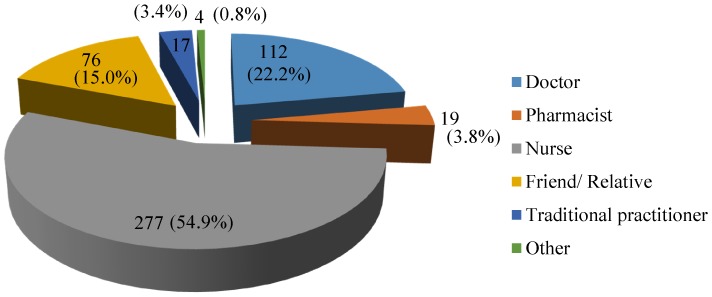
Distribution of individuals who administered injections to respondents.

In compliance with guidelines [31], most respondents engaged nurses as the main health professional for the administration of injections, followed by doctors. Of all respondents, 17 people stated traditional practitioners administered injections. About 15% of respondents reported that injections were administered by friends or relatives (Fig. 3).

Responses across different groups were significant by Kruskal- Wallis test [*H* = 11.1, df = 2, p = 0.004], administration of injections by nurses was more likely to have been to the older age group (more than 51 years) (Group 3: [*M* = 1.4, *SD* = 0.7]) than younger ones (range: 18–30 years) (Group 1: [*M* = 1.8, *SD* = 0.9]), p = 0.003.

### Respondents' attitude towards therapeutic injectable medicines

When all respondents were presented with questions regarding their attitudes toward injections, only seventy-seven respondents (16.2%) had an expectation of receiving injections in their mind when they visited a doctor. A significant difference was found using the Kruskal-Wallis test of the expectation of an injection across respondents in different age groups [*H* = 6.1, df = 2, p = 0.048], with respondents aged over 51 (Group 3: [*M* = 2.1, *SD* = 0.8]) being more supportive of the expectation than younger ones (range: 18–30 years) (Group 1: [*M* = 2.4, *SD* = 0.7]), p = 0.018. When respondents stated they preferred not to have injections prescribed, approximately one-third reported that doctors prescribed injections to them (n = 137, 29.0%).

When asked their opinion about therapeutic injections, 40% of all respondents agreed that injections were a better medicine (n = 190) than oral medications, and this was statistically significant between age groups [Kruskal- Wallis test, *H* = 18.5, df = 2, p<0.001]. Significantly older respondents (over 51 years) (Group 3: [*M* = 1.4, *SD* = 0.5]), agreed more with this statement when compared to younger respondents (Group 1: [*M* = 1.8, *SD* = 0.7, p<0.001] and (Group 2: [*M* = 1.7, *SD* = 0.7], p = 0.001). There were 63 (13.3%) of all respondents indicated injections were a better medicine with 221 (46.6%) who disagreed with this statement.

However, when all participants were asked for their opinions regarding treatment with injectable medicines, having an injection was not a personal preference for most respondents (n = 392, 82.7%) (Table 4).

**Table 4:** Reasons for injection preference.

Questions a	Yes	Sometimes	No
	n (%)	n (%)	n (%)
An injection helps you to recover faster	269 (56.8)	143 (30.2)	62 (13.0%)
An injection costs less	72 (15.2)	111 (23.4)	291 (61.4)
I prefer having an injection, because I forget to take medicines	126 (26.6)	108 (22.8)	240 (50.6)
When a doctor prescribes tablets/capsules, the treatment is more effective than injections	79 (16.7)	201 (42.4)	194 (40.9)
My friends, relatives recommend I have an injection	106 (22.4)	129 (27.2)	239 (50.4)
Medical companies advertise injections	103 (21.7)	118 (24.9)	253 (53.4)
Having an injection is a personal preference	22 (4.6)	60 (12.7)	392 (82.7)

a Some responses were missing for each category.

An important perception regarding injections was that they hastened the recovery process (n = 269, 56.8%) and a Kruskal-Wallis test yielded a statistically significant difference between age groups [*H* = 17.5, df = 2, p<0.001]. In particular, older respondents (over 51 years) (Group 3: [*M* = 1.2, *SD* = 0.5]) agreed with this statement more strongly when compared with respondents aged less than 51 years (Group 1: [*M* = 1.7, *SD* = 0.7]), p<0.001; (Group 2: [*M* = 1.6, *SD* = 0.7]), p = 0.004]).

However, more than half of the respondents agreed that treatment with oral medication was more or sometimes more effective than injections (n = 280, 59.1%). In general, most respondents did not support the statement that treatment cost was less with injections (n = 291, 61.4%) with younger respondents being significantly stronger in their disagreement than respondents older than 51 years [Kruskal- Wallis test, *H* = 12.4, df = 2, p = 0.002], (Group 1: [*M* = 2.5, *SD* = 0.7]), (Group 3: [*M* = 2.1, *SD* = 0.9]), p = 0.002.

Respondents also reported that when an injection was not prescribed that only 69 respondents (14.6%) would be disappointed and older respondents aged over 51 years [Kruskal-Wallis test, *H* = 20.8, df = 2, p<0.001] (Group 3: [*M* = 2.1, *SD* = 0.8]) were more likely to be disappointed if an injection was not received (p<0.001), (Group 1: [*M* = 2.6, *SD* = 0.7]), p<0.001; (Group 2: [*M* = 2.5, *SD* = 0.7]), p<0.001.

The questionnaire also asked if respondents would refuse therapeutic injections and 39.4% respondents (n = 187) answered they would refuse an injection if prescribed. Several reasons were proffered for refusing or rejecting injectable medicines (Table 5).

**Table 5:** Possible reasons for refusal if an injection was to be prescribed/supplied.

Reasons a	Yes	Sometimes	No
	n (%)	n (%)	n (%)
I am scared of pain	138 (29.2)	82 (17.4)	254 (53.4)
I am scared of needle injections	180 (38.1)	86 (18.2)	208 (43.7)
I do not trust doctors and pharmacists	46 (9.7)	141 (29.9)	287 (60.4)
It is possible to recover without any kind of injection	119 (25.2)	151 (32.0)	204 (42.8)
There are lots of dosage forms, e.g. tablets, capsules available for many diseases	129 (27.3)	126 (26.7)	219 (46.0%)
After sometime a disease cures by itself	48 (10.2)	124 (26.3)	302 (63.5)
There was no clean needle or syringe available	21 (4.4)	29 (6.1)	424 (89.5)
Others	126 (26.8)	52 (11.0)	296 (62.2)

a Some responses were missing for each category.

Of all participants only 22 males (11.3% of male cohort) and 19 females (6.8% of female cohort) had refused injections in the past. As the data in Table 5 demonstrate, the main reason for possible refusal was being scared of needles and injections (n = 180, 38.1%) and acknowledging the availability of other dosage forms than injections.

In particular, respondents aged between 18 and 30 years stated being scared [Kruskal-Wallis test, *H* = 8.7, df = 2, p = 0.013], (Group 1: [*M* = 2.1, *SD* = 0.9]) compared with those aged over than years 51 (Group 3: [*M* = 2.5, *SD* = 0.7], p = 0.013). Similarly, younger respondents [Kruskal-Wallis test, *H* = 12.1, df = 2, p = 0.002] were likely to accept that other dosage forms, including tablets, capsules and other administration forms were available (Group 1: [*M* = 2.1, *SD* = 0.8]); (Group 3: [*M* = 2.5, *SD* = 0.8]), p = 0.002.

In general, most respondents trusted their doctors and pharmacists. In addition, most did not support that after a period of time a disease would be cured by itself (n = 302, 63.5%).

## Discussion

This is a part of a larger study that has assessed the prescribing practice of antibiotics, including injections for mild/moderate community-acquired pneumonia (CAP) in Mongolia [32]. Community members' views, attitudes, knowledge and experiences regarding the prescribing of injections were analysed in this study.

A high level of injection prescribing was evident in this study. More than half (56.7%) of the respondents, which were an ambulatory cohort of the public, had received injection(s) almost always for the treatment of an illness in the past 12 months. Their injection exposure amounted to 1891 per 1000 of the total sample or almost 2 injections per person per year. Additionally this cohort was younger and presumably therefore healthier and also less predisposed towards injections than would be a population representative sample. Complying with current regulations, injections were frequently prescribed by a doctor and supplied from a pharmacy. However, doctors were found to be both prescribing and supplying injections in Mongolia. This indicates that doctors are a major contributor to the high level of injection use in Mongolia. The high number of doctors in Mongolia [23] may be a contributing factor since the prescribing of injections can provide repeated consultations. Some the prescribing and supply of injections was also carried out by individuals other than specified in the regulations. Inappropriate prescribing of injections by nurses and pharmacists should be ceased. Some countries with comparable health systems have also reported high levels of inappropriate prescribing and injection use [4], [10], [26], [33]. The high numbers of doctors with respect to the population is however unique to Mongolia.

In the past decade, little has been reported regarding the perceptions and attitudes of patients towards injections. Past literature has suggested that patients are often one of the main drivers that fuel the inappropriate use of injections [10], [26], [34]–[38]. In contrast, this study found that only a minority of community members (16%) always/often expected injections to be prescribed. From those who expected injections, older people tended to expect injections for common medical conditions and this reflects other findings [39]–[41]. It is unknown whether the younger age groups' dislike of injections is a preference that changes with ageing or the current younger generation will become a long standing barrier to injection prescribing. There is clear evidence of the respondents understanding of the need for clean needles and syringes predicated by HCV which maybe a contributing factor to their dislike. Other studies, have also reported a high level of awareness of using new syringes and needles for injection use [37], [42]. However, it is also possible that a high awareness of the associated risks of unsafe injection practises can be a cause for an increased utilization of disposable syringes and needles instead of alternative forms of treatment.

In Mongolia, community members indicated injections hastened the recovery process and this was consistent with other findings [26], [37], [38]. In addition, some community members in this study indicated that injections were a better medicine than oral medications (n = 190, 40%) and this confirmed a previous finding from Mongolia [14].

Health workers in developing countries have reported that community member's compliance was improved with injections than with oral medication [2], [11] and similarly, doctors and pharmacists in a questionnaire study administered as part of this overall study indicated choosing an injection was to often avoid non-compliance problems [32], [37], [38]. It is clear that the respondents in this study would not choose an injection as an option to improve compliance.

In addition to the formal administrators (for example: nurses and doctors), pharmacists and friends/relatives were identified by the respondents as injection administrators. Similarly, studies in Egypt and India reported that unqualified medical providers, including relatives, housekeepers of government clinics and assistants of private medical doctors often administered injections [43]. Reasons for choosing unqualified medical providers were explained by their availability and accessibility at low or without any extra cost [35] [43]. There are public health issues with unqualified practitioners administering injections.

### Limitations

The selection of community members was not random, however the response rate of community members was high (79%). The study aimed to recruit community members from various socioeconomic groups, by administering the questionnaire at 55 different regions of Ulaanbaatar, shopping centres, hospitals and pharmacies that were located in the central and semi-rural parts and different socioeconomic areas. However, some differences were apparent in demographic characteristics of respondents compared with the general population. It is also possible that the responses from community members could be influenced by issues of social desirability. The questionnaires were however, anonymous and confidentiality was emphasized encouraging honesty. Some of the questions were based on recall of events which may not always be complete. Factual questions however related to injections administered in the last year to limit this factor. Forms were assessed for completion by the researcher to improve completion. It is possible that those who did not volunteer may have had different views. Although the respondents were not the same as the population the main underrepresented group was the older age cohort and more likely to support the administration injections. Some caution must be exercised in generalising the findings to the whole population.

## Conclusion

These findings suggest high levels of inappropriate use of injections occurred in Mongolia. The current high level of medical prescribing and supply of injections is a significant potential public health hazard in Mongolia. Illegal provision of injections by pharmacists and other health practitioners should be eliminated. Intervention campaigns addressing issues regarding appropriate prescribing and use of injections should be implemented for prescribers. Further research is needed to assess the proportion of administered injections that are unnecessary and hence could reduce the public health hazard in Mongolia.
